# Development of alkyd-based copper sorbate coatings for anti-corrosion and anti-biofouling applications

**DOI:** 10.3389/fchem.2026.1838832

**Published:** 2026-09-30

**Authors:** Manikandan Thiyagarajan, Subramania Angaiah, Chao Yan, Parthipan Punniyakotti

**Affiliations:** 1 School of Materials Science and Engineering, Jiangsu University of Science and Technology, Zhenjiang, China; 2 Electro-Materials Research Laboratory, Centre for Nanoscience and Technology, Pondicherry University, Puducherry, India; 3 Department of Biotechnology, Faculty of Science and Humanities, SRM Institute of Science and Technology, Chengalpattu, Tamil Nadu, India

**Keywords:** alkyd paint, anti-corrosion, biofouling, copper sorbate, electrochemical impedance spectroscopy

## Abstract

**Introduction:**

Biofouling is a serious problem in various industrial sectors such as shipping infrastructure, cooling water systems, and aquaculture. Biofilm formation by different types of microorganisms plays a very crucial role in this biofouling process. Biofouling on metallic surfaces accelerates aggressive corrosion processes. Therefore, it is essential to develop antifouling coatings with corrosion resistance for effective protection.

**Methods:**

In the present study, copper sorbate (CS) and copper oxide (CuO) were synthesized as antifouling pigments and characterized using a UV-vis spectrometer, Fourier transform infrared spectroscopy, X-ray diffraction, and field emission scanning electron microscopy. Both pigments in different ratios were used for the formulation of antifouling and anti-corrosion paints. Salt spray test and anti-corrosion test were performed in the presence of 3.5% NaCl solution for 30 days. Electrochemical impedance spectroscopy (EIS) and potentiodynamic polarization were recorded for all the tested specimens.

**Results and Discussion:**

The results indicate that CS-rich coatings (15%20% of CS) display higher corrosion resistance than CuO-rich coatings. The antifouling potential of the coated mild steel was evaluated by immersing them in the marine estuarine water, and surface changes were monitored at regular intervals. All coatings exhibited considerable antifouling effects up to 60 days of immersion. Notably, the CS-II coating, containing only 5% CuO with 15% of CS, showed significant fouling resistance up to 90 days. At the end of 120 days, SEM analysis confirmed that a huge number of marine microorganisms (mostly diatoms) settled on CS-I, CS-III, CS-IV, and CS-V, but minimal microorganisms were only noticed on CS-II. This could be due to the antimicrobial activity and controlled leaching behavior of both pigments in marine conditions.

## 1 Introduction

Marine biofouling begins with the attachment and colonisation (biofilm) of the free-floating marine microorganisms, such as bacteria and microalgae, onto any marine substrate, followed by the settlement of the macro-organisms, such as molluscs, oysters, and barnacles (Ni et al., 2026; Ye et al., 2019). Fouling issues are not only found in the marine sectors, but they are also causing major issues to domestic water transportation facilities as well as in purification sectors, power plants, and drainage systems (Chakraborty et al., 2026; Lei et al., 2026). The biofouling problem makes a huge negative impact on economic, safety, and environmental aspects in the maritime industry by causing undesired damage to marine vehicles, unanticipated biological attack on marine structures, and huge fuel consumption (Chen X. et al., 2025; Dai et al., 2019; Shang et al., 2020). A persistent fouling problem on submerged metal structures leads to marine corrosion problems, including microbial corrosion (Chen G. et al., 2025; Sivakumar et al., 2025).

Repairing or replacing the affected parts makes fouling issues one of the key problems in the maritime industry. To overcome fouling problems, many methods are being implemented, such as using antifouling paints, coatings with organic and inorganic pigments, biocide-based paints, polymer-based coatings, nano-composite coatings, and foul release coatings (Al-Busaidi et al., 2025; Zan et al., 2024; Zhang et al., 2018). Organotin-based pigments actively prevent fouling problems, but their toxicity against most of the marine non-fouling organisms has led to restrictions on their use in the maritime industry. Additionally, their abundance in marine water provides a route for entering other animals through the food chain. Till now, conventional coatings have been prepared with copper oxide (CuO) as antifouling compounds, which are also considered toxic compounds for many of the marine organisms. Replacing or reducing the CuO concentration is one of the primary intentions of the researchers who are working on the development of antifouling coatings (Huang et al., 2026; Mars et al., 2025). Because using these antifouling technologies saves billions of dollars per annum in the shipping industry alone (Ma et al., 2017).

Many of the natural antifouling compounds are being evaluated for antifouling properties from marine sources such as algae, sponges, corals, and tannins from plant sources (Adouane et al., 2026; Bae et al., 2026; Zhao et al., 2026). These natural antifouling compounds show considerable antifouling properties, but the problem associated with using marine species is their low yield and scale-up challenges. Hence, finding alternative eco-friendly anti-fouling compounds with a non-toxic nature is one of the primary tasks for researchers to overcome the fouling problem in the marine industry.

A few researchers have identified that sorbate can act against the fouling organisms. Potassium sorbate is a commonly used salt of an unsaturated short-chain fatty acid (Parsons et al., 2012). Potassium sorbate is widely used in the food industry as a preservative to prevent the growth and development of a variety of microorganisms, such as bacteria, fungi, mold, and yeast. Remarkably, sorbate-based compounds were non-toxic to the non-targeted organisms (Pérez et al., 2014). In addition to these characteristics, potassium sorbate is an eco-friendly chemical compound and is classified as a safe chemical by the US Food and Drug Administration (Plumridge et al., 2008). Hence, using the sorbate-based materials for the prevention of fouling problems is categorised as the safest as well as the most eco-friendly approach.

The highly soluble nature of potassium sorbate in water is one of the major drawbacks in its use as an antifouling compound. This makes it easily leachable while making antifouling paints, which in fact protect the coated substrate in submerged conditions. Further, this leaching leads to a negative impact by creating blistering and pores over the coating (Jamsa et al., 2013). To overcome this leaching issue and also to extend their life in submerged conditions, sorbate-based pigments are prepared with metallic cations. Very few metallic sorbate materials, such as aluminium sorbate and ferric sorbate, are prepared, which show considerable anti-fouling activity (Pérez et al., 2014). Still, many metal-based sorbate materials need to be found as efficient and non-toxic antifouling materials.

The present study focuses on the synthesis and characterisation of copper sorbate (CS) as an antifouling pigment. Different concentrations of CS pigment with biocidal capability and CuO (as an established antifouling pigment for comparison) were used for the formulation of the anti-fouling paints with alkyd resin as a binder. In this study, alkyd resin was preferred over other types (e.g., acrylic) because of its film-forming characteristics, outstanding adhesion, elasticity, low-cost, and compatibility with copper-based pigments. Formulated paints are characterised both physically and mechanically to find out their fineness and additive natures. In general, many researchers consider only the antifouling capability of coatings, which sometimes leads to system failure due to the corrosion problems associated with immersed environments. To overcome these problems, prepared paints are coated on the mild steel 1010 (MS) specimens and subjected to laboratory anticorrosion experiments, including salt spray and electrochemical (both AC impedance and potentiodynamic polarisation) analysis. Further, all the coated specimens were subjected to the marine immersion study in the estuary water for 120 days. At the end of the fieldwork, specimens were subjected to surface analysis with scanning electron microscopy.

## 2 Materials and methods

### 2.1 Materials

Potassium sorbate, aluminium stearate, soya lecithin, and cobalt naphthenate were purchased from Otto Chemie Pvt Ltd., India. Xylene, copper (II) chloride, sodium hydroxide, sodium chloride, potassium chloride, disodium hydrogen phosphate, and potassium dihydrogen phosphate were purchased from Hi-media, India. Alkyd resin was received from Aroma Chemical Agencies, Chennai. All the chemicals were used as received without any further purification. Mild steel specimens were purchased from a local merchant shop.

### 2.2 Synthesis of CuO

Copper oxide (CuO) was synthesised by a simple co-precipitation method as described in Phiwdang et al. (2013). Briefly, a 400 mL of 0.5 M aqueous NaOH solution was placed in a beaker and kept in a magnetic stirrer while stirring at 80 °C. To this solution, a 100 mL of 1 M aqueous CuCl_2_ solution was added drop-by-drop to get a block colored precipitate, which indicates the formation of CuO. Finally, the CuO was filtered using No.1 Whatman filter paper and washed several times with deionised water and absolute ethanol, and dried in a vacuum oven for 6 h at 80 °C.

### 2.3 Synthesis of copper sorbate pigment

Copper sorbate (CS) pigment was prepared by using a modified procedure as described by Perez et al. (2014). For synthesis, a 100 mL of 0.3 M of aqueous CuCl_2_ solution was dropped into 60 mL of 1 M of potassium sorbate solution under continuous stirring at 80 °C to get CS as a precipitate. It was filtered using No.1 Whatman filter paper and then washed several times to remove unreacted content from the pigment. Finally, the obtained CS was dried in a vacuum oven for 12 h at 80 °C.

### 2.4 Preparation of marine anti-fouling paints

Anti-fouling paints were prepared as follows: The paint components and their concentrations are clearly mentioned in Table 1. For various anti-fouling paint formulations, initially, alkyd resin (binder) was taken at about 32%, and 40% of solvent was added under stirring conditions for 1 h. The pigment volume concentrations (PVC) were set as 20%, a primary pigment CS wt% as 20 (CS-I), 15 (CS-II), 10 (CS-III), 5 (CS-IV), and 0 (CS-V), along with known anti-fouling pigment, copper oxide with 0, 5, 10, 15, and 20%, respectively. Both pigments (CS and CuO) were added to the above mixture and then stirred for 2 h. Finally, the additives such as wetting agent, anti-settling agent, and naphthenate-based curing agent were added and stirred for 30 min. The obtained paints were stored in airtight containers for further use.

**TABLE 1:** Components and their roles in antifouling paint formulation.

Component (role)	CS-I (%)	CS-II (%)	CS-III (%)	CS-IV (%)	CS-V (%)
Copper sorbate (pigment)	20	15	10	5	-
Copper oxide (pigment)	-	5	10	15	20
Alkyd resin (binder)	32	32	32	32	32
Xylene (thinner/solvent)	40	40	40	40	40
Aluminium stearate (anti-settling agent)	3.2	3.2	3.2	3.2	3.2
Soya lecithin (wetting agent)	3.2	3.2	3.2	3.2	3.2
Cobalt naphthenate (drier)	1.6	1.6	1.6	1.6	1.6

### 2.5 Preparation of coatings and physical characterization

#### 2.5.1 Preparation of specimens

Mild steel specimens were prepared with different dimensions for different purposes, such as 25 mm × 75 mm for salt spray and electrochemical studies, 100 mm × 150 mm × 0.8 mm for the conical bend test, and 50 mm × 75 mm for adhesion and field immersion studies. All the specimens were subjected to mechanical polishing with 200–600 mesh silicon carbide papers. Further, all the specimens were surface cleaned with deionised water, followed by acetone, and dried at room temperature.

The fineness of all the prepared paints was tested using the TQC Grindometer (Model: VF2111, Make: Sheen, Netherlands) according to ASTM D1210. Further, different paints were coated on the surface-polished MS specimens using the conventional brush coating method for different purposes. After making the coatings, specimens were kept at room temperature for 24 h for complete drying purposes. Thicknesses of the coatings were measured using a digital thickness gauge. Flexibility or elasticity of the painted specimen to elongation from the coated substrate was performed with the help of a conical mandrel according to the ASTM D522 standard. The adhesion property of the coating was confirmed by the cross-hatch cutter by making a cross-cut over the coated substrate, then the adhesive tape was applied over the cross-cuts, and then the adhesive tape was pulled out to observe the adhesive nature of the paint, as per ASTM D3359-B.

#### 2.5.2 Characterization

The optical properties of the synthesized pigments were analysed using a UV-Vis Spectrophotometer using Shimadzu, UV 3600 Plus. The vibrational properties of the synthesized compounds were studied using a JASCO-4600 Fourier transform infrared (FT-IR) spectrometer. The FT-IR spectra were recorded in the range of 400-4000 cm^-1^ using KBr pellets at a spectral resolution of 4 cm^-1^. Further, the crystalline structure of CuO and CS nanoparticles was detected by means of X-ray diffraction (XRD) analysis using a Rigaku-Ultima IV X-ray diffractometer operated at 40 kV and 40 mA with nickel filtered Cu- Kα radiation (λ = 0.154 nm) at a measurement rate of 2° min^-1^. The surface and elemental analysis of the synthesized pigments was done by Field Emission Scanning Electron Microscopy (FESEM) using JEOL IT800HL. In addition, the surface morphology of the paint coating on an MS (10 × 10 mm) was carried out by using scanning electron microscopy (Hitachi, Model S-3400N).

### 2.6 Anti-corrosion studies

For the anti-corrosion study, all the paint-coated MS surfaces were prepared with an exposure area of 1 cm^2^ with the help of cylindrical tubes, and 15 mL of 3.5% of NaCl solution was poured into the exposure areas and kept at room temperature for 30 days. The electrolyte loss due to evaporation was recharged with deionized water to maintain the fixed volume and concentration throughout the study period. After 15 days of immersion, electrochemical analysis such as AC impedance and potentiodynamic polarisation studies were carried out using a three-electrode system with Ag/AgCl as the reference electrode, Platinum as the counter electrode, and a paint-coated surface with 1 cm^2^ as the working electrode. After maintaining a stable open-circuit potential (OCP), impedance measurements were carried out in the frequency range of 100 kHz to 10 mHz with an AC amplitude of 5 mV. Potentiodynamic polarisation was recorded in the polarisation range of ±250 mV at a scan rate of 1 mV/s with respect to OCP. All the electrochemical analyses were performed using an electrochemical workstation (Biologic, Model: VSP, France). Similar observations were recorded after the 30^th^ day of immersion. The entire test was done in triplicate.

### 2.7 Salt spray test

To confirm the anti-corrosion aptitude of the coated specimens in a marine environment, a salt spray test was carried out as specified in the standard ASTM B117. A cross-cut towards the end-to-end was made on the coated specimens for electrolyte migration. 3.5% NaCl was prepared and continuously sprayed (fog) in the salt spray chamber using compressed air. Their surface changes were monitored continuously.

### 2.8 Anti-fouling studies

A rectangular wooden raft was prepared to hang the entire coated specimen, and the tied specimen raft was immersed in the Vellar estuary near Parangipettai, Tamil Nadu, India. This estuary was the best place to do an antifouling field study, because the pH of the water was maintained around 8.2, salinity was recorded as 28–30 ppt, and many of the micro (bacteria, diatoms) and macro-fouling organisms were reported as predominant phytoplankton and zooplankton groups in this estuary. Samples were immersed 1 m below the water surface for 120 days. Surface changes during the immersion period were monitored at 30-day intervals, and digital images were taken with the aid of a Nikon D-5300 DSLR camera. The entire study was done in triplicate.

### 2.9 SEM analysis

At the end of the immersion study, the fouling nature was monitored using SEM analysis. The immersed paint-coated specimens were taken out on the 120^th^ day (micro-fouling observed) and fixed with 3% of glutaraldehyde in phosphate-buffered saline (PBS) (pH: 7.4) for 4 h. The specimens were then washed with PBS for two times with a 5 min interval and then washed with deionised water, followed by dehydration with 50, 75, 95, and 99% ethanol; each step was allowed to remain for 10 min. Finally, the coupons were dried and examined through SEM analysis (Rajasekar et al., 2017).

## 3 Results and discussion

### 3.1 Pigment characterization

The UV-visible spectrum of synthesized CuO and copper sorbate is provided in Figure 1. A broad absorption peak was noticed in the UV region (240–280 nm) for the synthesized CuO, which was further extended into the visible region. Similarly, copper sorbate exhibited a broad absorption peak in the UV range, which indicates the characteristic electronic transitions linked with the sorbate moiety and Cu^2+^ coordination. This observation confirms the successful formation of CuO and copper sorbate (Khatoon et al., 2023).

**FIGURE 1:**
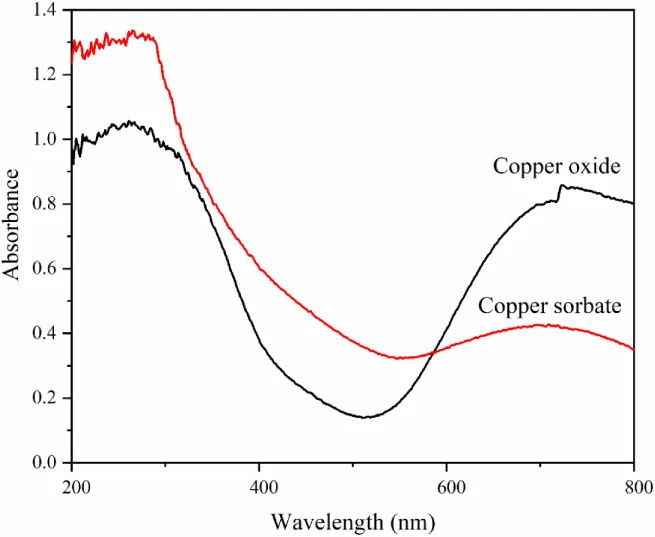
UV-Visible spectrum of synthesized copper oxide and copper sorbate pigments.

The XRD pattern of CuO is presented in Figure 2a. From this figure, it is clear that the obtained CuO was crystalline in nature. The obtained crystalline peaks were possibly indexed as the monoclinic phase of tenorite with space group C2/c, which was consistent with the JCPDS No. 65–2309 (Venkateswaran et al., 2010). The major peaks observed at 35.61° and 38.83° correspond to (−111) and (111) planes, respectively. The other small peaks were observed at 32.50°, 48.83°, 53.55°, 58.32°, 61.69°, 66.27°, 68.12°, 72.19° and 75.19 matching to (110), (−202), (020), (202), (−113), (−311), (220), (311), and (044) crystalline planes of CuO, respectively. The synthesised CuO was free from other oxides or impurities such as Cu_2_O/Cu_3_O_4,_ which was confirmed by the phase purity of CuO (Sreeju et al., 2019). Similarly, the XRD spectrum obtained for the synthesised Cu-sorbate pigment is shown in Figure 2b, which confirms its crystalline nature. As shown in Figure 2b, strong peaks were observed in the 10°–30° range. Within this range, sorbic acid peaks were observed around 25° and 28° (Pang et al., 2015). A copper peak was observed around 43°–44° (Nasrollahzadeh et al., 2017). This observation confirms that CS was formed successfully by the co-precipitation method.

**FIGURE 2:**
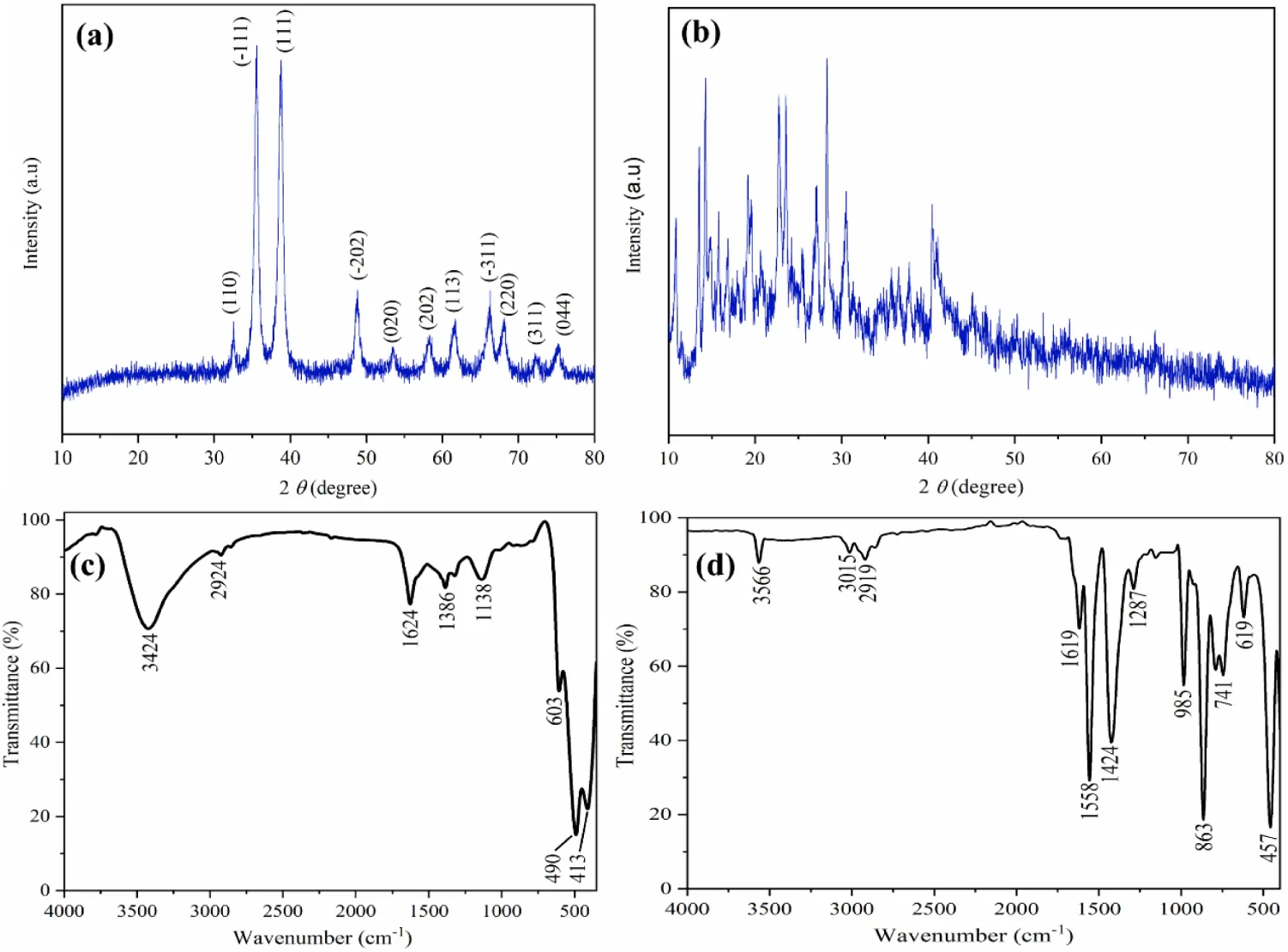
**(a,b)** XRD pattern, and **(c,d)** FT-IR spectrum of synthesized copper oxide and copper sorbate, respectively.

The functional groups present in CuO were confirmed by the FT-IR spectrum, as mentioned in Figure 2c. A broad band at 3424 cm^-1^ was the characteristic peak of O-H stretching vibrations due to the surface-adsorbed water molecules. Peaks found between 1,383 and 1,624 cm^-1^ were assigned to O-H bending vibrations combined with copper atoms (Phiwdang et al., 2013; Vishveshvar et al., 2018). The peak at 1,138 cm^-1^ belongs to the C-O vibration due to the adsorption of atmospheric CO_2_. Peaks present at 603, 490, and 413 cm^-1^ strongly confirm the stretching and lattice vibrational modes of the Cu-O chemical bond of CuO with monoclinic structure (Assaouka et al., 2022; Phiwdang et al., 2013; Uma et al., 2021). These observations confirm that the CuO formed effectively.

Further, the FT-IR spectrum obtained for the synthesized copper sorbate is shown in Figure 2d. The bands at 3015 and 2,919 cm^-1^ correspond to olefinic and aliphatic C–H stretching, respectively. The band at 1,619 cm^-1^ was attributed to the C=C stretching of the conjugated sorbate chain. The bands at 1,558 and 1,424 cm^-1^ belonged to the asymmetric and symmetric stretching vibrations of the carboxylate (COO^−^) group, indicating their interaction with Cu^2+^. A band at 457 cm^-1^ was attributable to Cu-O vibration, which confirmed the formation of copper-sorbate coordination (Abousaty et al., 2024).

Further, the synthesized CuO and copper sorbate are subjected to surface analysis using FESEM as illustrated in Figure 3. From Figures 3a,b the synthesized CuO appeared as agglomerated particles with an irregular, rough surface morphology, which was common in the co-precipitation synthesis due to the high surface energy. Similarly, Figures 3c,d shows the FESEM images of synthesized copper sorbate with elongated, needle-like crystalline structures that are densely packed and randomly oriented. This morphology highlights a rough, fibrous surface texture, suggesting high surface area and distinctive microstructural features. Further, elemental analysis of both CuO and copper sorbate is illustrated in Figure 4. The EDS spectrum and table of both pigments (Figures 4a,b,g,h) confirm the balanced distribution of each element. A peak around 1.5 KeV was belongs the Aluminium, which was used for the loading of the sample for analysis. In addition, elemental mapping of CuO (Figures 4c,d,e,f) and CS (Figures 4i,j,k,l,m) clearly shows the uniform distribution of each element in the synthesized pigments.

**FIGURE 3:**
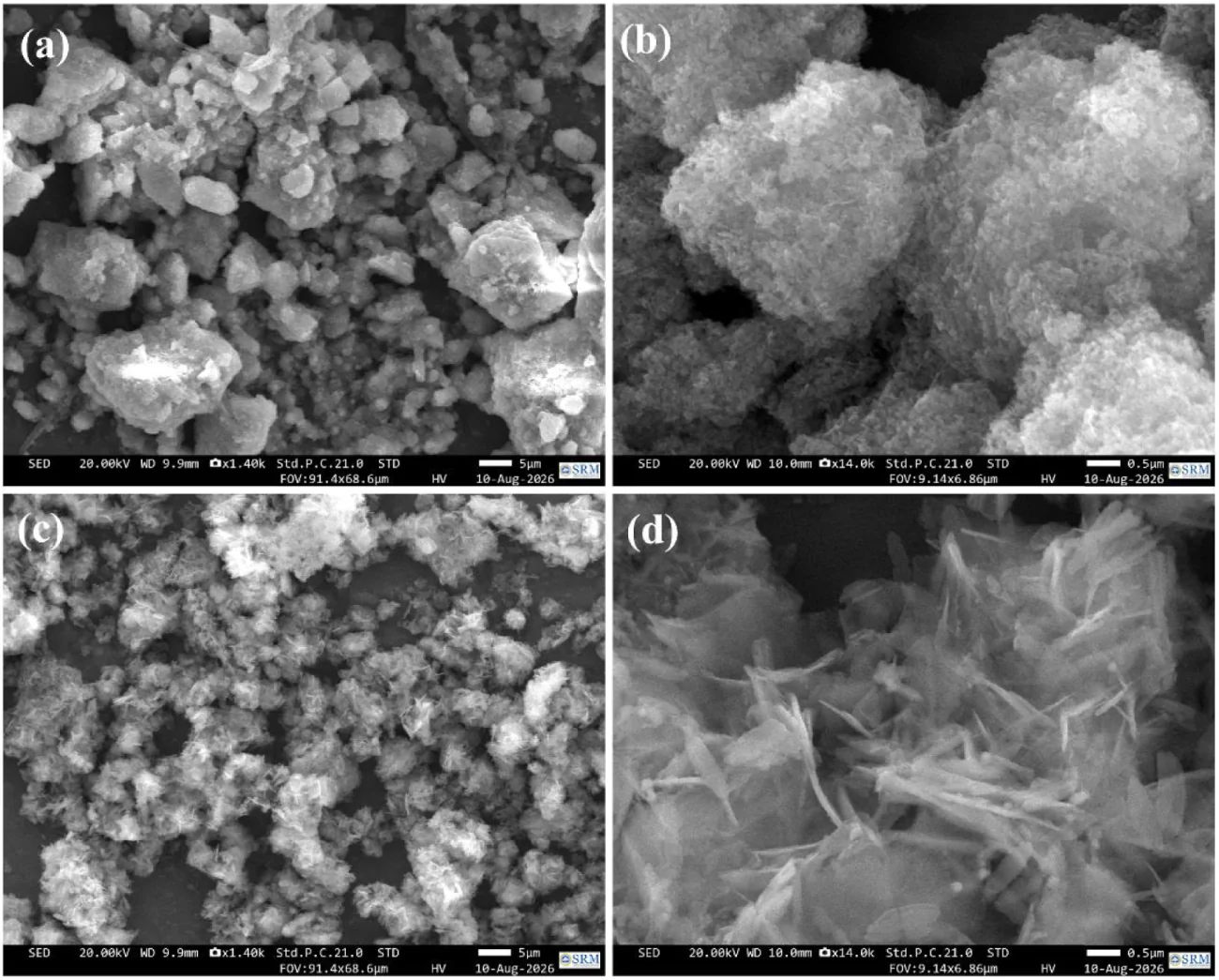
Field emission scanning electron microscopy analysis: **(a,b)** chemically synthesized CuO; and **(c,d)** copper sorbate at low and higher magnification, respectively.

**FIGURE 4:**
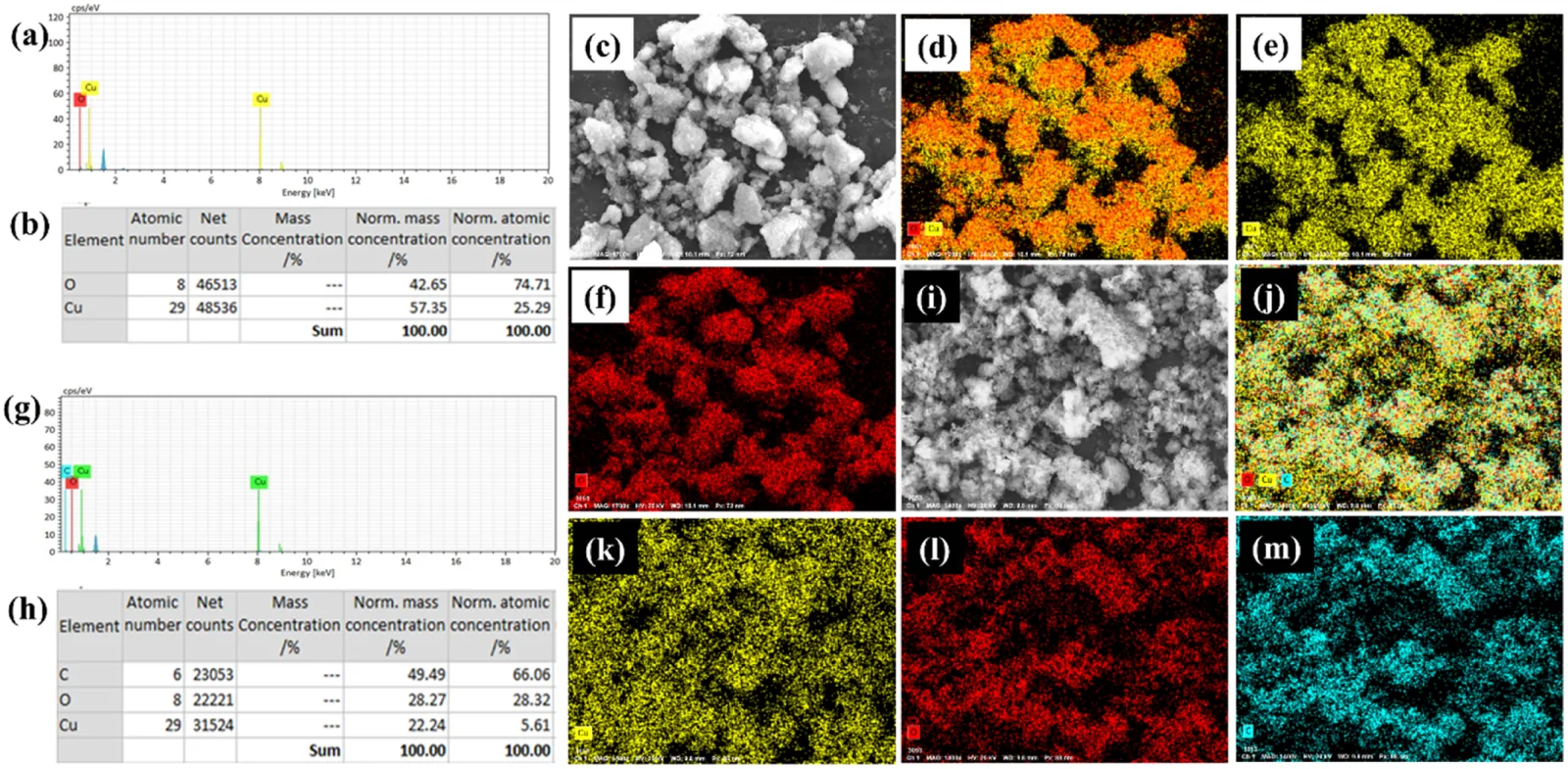
Elemental analysis of the synthesized pigments: **(a,b)** EDS spectrum and table, **(c–f)** Elemental mapping of copper oxide (Cu and O), respectively; **(g,h)** EDS spectrum and table, **(i–m)** Elemental mapping of copper sorbate, respectively.

### 3.2 Physical properties of the paint

All the formulated paint-coated specimens were subjected to different physical characterizations such as thickness, fineness, adhesion to substrate, and flexibility test, as mentioned in Table 2. The thicknesses of different coatings were in the range of 214 μm–510 µm. The coatings were fabricated using a manual application process, which may have resulted in minor thickness differences despite maintaining identical preparation and curing conditions. Fineness of the formulated paints was confirmed with a grindometer, and their fineness was within the range of 5–15 ± 2.5 µm, with the highest fineness of 5 ± 2.5 µm being recorded for CS-I and the lowest for CS-III and CS-IV, respectively. This fineness test confirms that formulated paints were very finely ground (Supplementary Figure S1). Further, all the paints were subjected to the adhesion test; from this observation, it was clear that all the paints had good adhesion with the substrate, which means no peel-off was found in any cut areas or edges (Supplementary Figure S2). The conical mandrel bend test was carried out to find the flexibility of coated specimens. While making bends, for this purpose, a thickness of 0.8 mm of MS was used. From the bend test (Supplementary Figure S3), no cracks or fragility were noticed in any of the coating systems. It shows that the formulated paints have good adhesion as well as mechanical strength even in the bent condition.

**TABLE 2:** Physical characterisation of formulated paints and their corresponding coatings.

Paint	Thickness (µm)	Fineness			Adhesion strength	Bend test
		H	PCU	µm		
CS-I	510 ± 0.02	8.0 ± 0.25	10.0 ± 0.25	5 ± 2.5	5B	Pass
CS-II	221 ± 0.02	7.75 ± 0.25	8.5 ± 0.25	10 ± 2.5	5B	Pass
CS-III	214 ± 0.015	6.75 ± 0.25	8.25 ± 0.25	15 ± 2.5	5B	Pass
CS-IV	316 ± 0.02	6.5 ± 0.25	8.25 ± 0.25	15 ± 2.5	5B	Pass
CS-V	241 ± 0.02	7.0 ± 0.25	8.75 ± 0.25	10 ± 2.5	5B	Pass

### 3.3 Surface studies of the paint-coated specimens

All the paint-coated specimens were subjected to surface analysis under a scanning electron microscope (SEM) to explore the surface nature as described in Figure 5. From the SEM analysis, it was very clear that the coated specimens were very smooth, and the coatings were uniformly made on the specimens. Particularly, for the paints CS-I to CS-IV, a uniform surface was observed, and smoothness decreased with increasing CuO concentration, which could be due to their heterogeneous nature.

**FIGURE 5:**
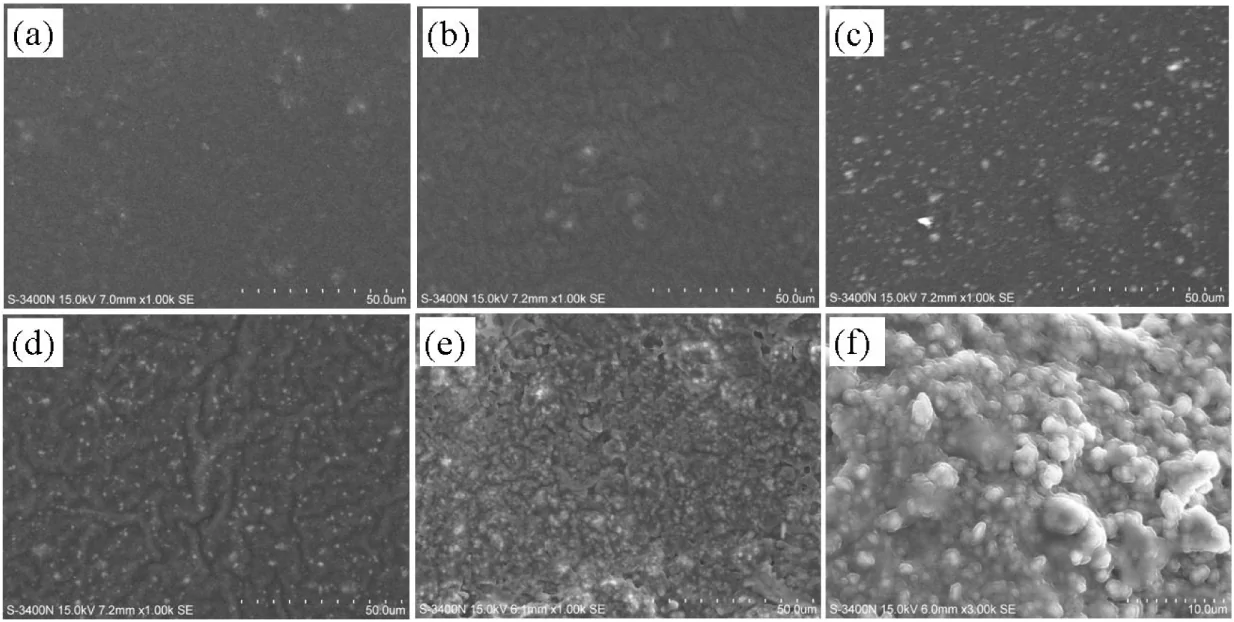
SEM analysis of coated specimens **(a)** CS-I, **(b)** CS-II, **(c)** CS-III, **(d)** CS-IV, **(e)** CS-V, and **(f)** higher magnification of CS-V.

### 3.4 Anti-corrosion studies

#### 3.4.1 Electrochemical impedance studies

Anti-corrosive performance of all the coated specimens was studied in 3.5% NaCl solution for 30 days with the help of an electrochemical impedance analyser (EIS). The results obtained from EIS studies are presented in Table 3 and Figure 6. Impedance analysis was measured after 15^th^ day (Figure 6a) and 30^th^ day (Figure 6b) of immersion to monitor the continuous changes in the corrosion resistance during the incubation period. From Figure 6a, clearly illustrated that a small semi-circle was present in bare MS, and this semi-circle was massively increased with decreasing CuO content, and the largest semi-circle was observed for CS-II. It means that there were higher corrosion protection properties in Cu-sorbate-rich paint (15% CS + 5% of CuO). At the same time, the electrochemical result of this study indicates that increasing CuO content beyond the optimum level decreased the anticorrosion capacity of the coating, which is attributed to a decrease in coating resistance and barrier integrity during seawater exposure rather than to the intrinsic nature of CuO, as confirmed in the SEM. Moreover, the Bode plots (Figure 7) and phase angle plots (Figure 8) further support the observations obtained from the Nyquist impedance diagram (Lu et al., 2017). As illustrated in Figure 8, the one-time constant of the phase angle plot strongly suggests the barrier capacity of the coating. Furthermore, the resistance was higher in CS-II, followed by CS-I and CS-III, when compared to other coatings. After the 15^th^ day of immersion, the original charge-transfer time constant was shifted towards the intermediate frequency range (4.5–5.5 Hz), followed by the appearance of a third time constant in the lower frequency range (1–3 Hz) in the phase angle plots of CS-IV and CS-V coatings. This was due to the diffusion of corrosive electrolyte into the coating and also due to the rust formation on the coating or interface layer (Murillo-Gutiérrez et al., 2013).

**TABLE 3:** Electrochemical impedance analysis of different anti-corrosive paints coated specimens immersed in 3.5% NaCl at different time intervals.

Paint system	Day	R_s_ (Ω. cm^2^)	C_dl_ (F. cm^2^)	R_ct_ (Ω. cm^2^)
CS -I	15	133.1	7.925 × 10^−9^	467
	30	144	4.703 × 10^−9^	297.5
CS-II	15	577.8	0.407 × 10^−9^	11,929
	30	141.7	7.577 × 10^−9^	532.6
CS-III	15	208.6	27.1 × 10^−9^	139.3
	30	48.01	19.8 × 10^−9^	39.32
CS-IV	15	97.22	53.09 × 10^−9^	89.64
	30	42.14	0.274 × 10^−6^	19.83
CS- V	15	37.19	0.852 × 10^−9^	17.31
	30	27.46	0.423 × 10^−6^	9.678
Bare	15	11.19	0.238 × 10^−3^	0.368
Specimen	30	11.29	0.270 × 10^−3^	0.3586

**FIGURE 6:**
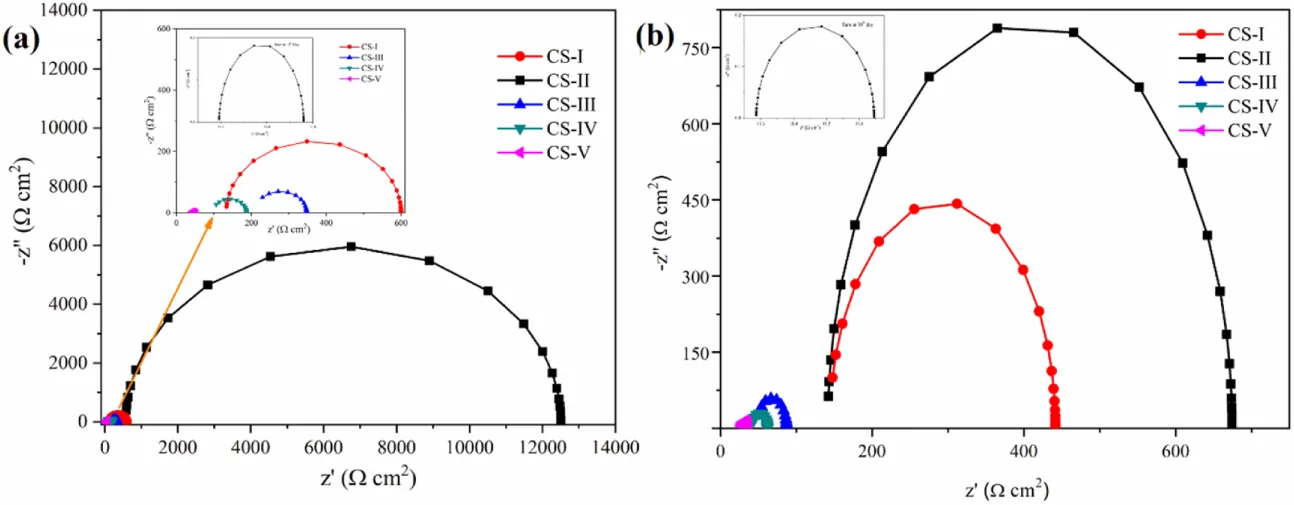
Nyquist plots of different anti-corrosive paint-coated specimens at **(a)** day 15 and **(b)** day 30.

**FIGURE 7:**
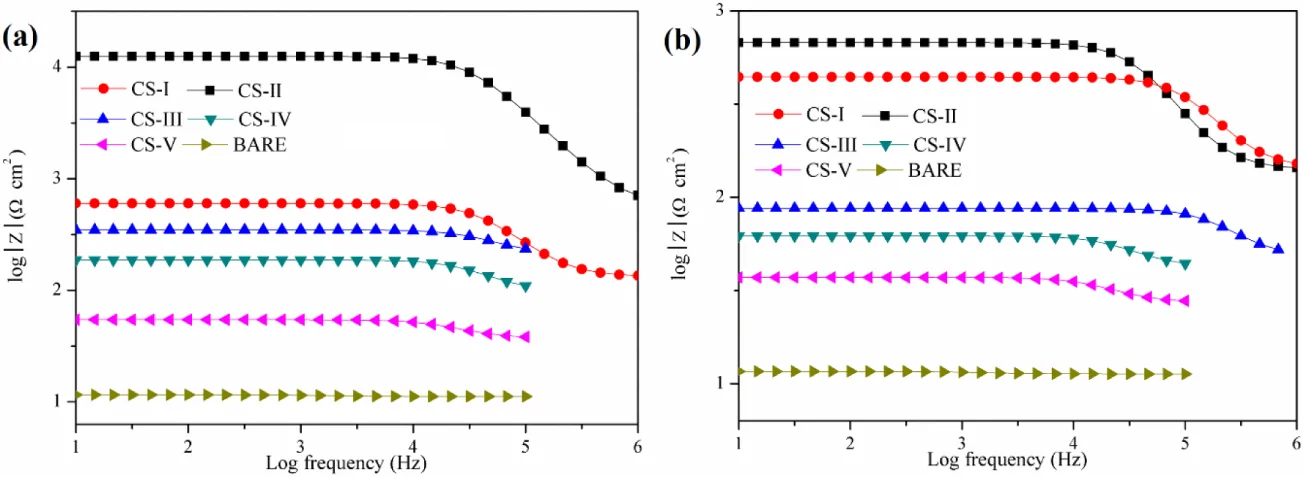
Bode plots of different anti-corrosive paints coated specimens at **(a)** day 15, and **(b)** day 30.

**FIGURE 8:**
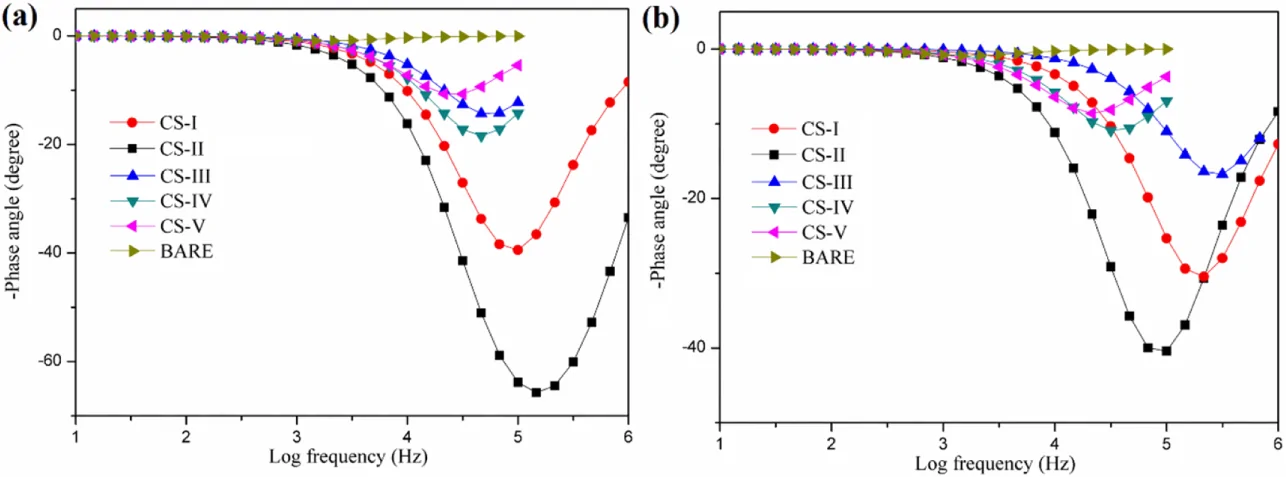
Phase angle plots of different anti-corrosive paints at **(a)** day 15 and **(b)** day 30.

The results presented in Table 3 indicate the high corrosion resistance of all paint systems for the first 15 days, compared to the bare MS. The highest charge transfer resistance (*R*_*ct*_) of 11,929 Ω. cm^2^ was determined for the CS-II sample. However, further increase of CuO content in the paint formulations significantly reduced the *R*_*ct*_ value.

The coating degradation rate slowly increased with increasing immersion period for all the systems (Figure 6b). This may be due to the chloride ions reacting with the coating surface, which may influence the chemical stability of the coating. However, in the CS-II system (with 15% of copper sorbate), an effective corrosion resistance was observed until the 15^th^ day, which dropped to 532.6 Ω. cm^2^ after the 30^th^ day of immersion. Between the 15^th^ and 30^th^ day of the incubation period, the *R*_*ct*_ value fell more than 20-fold. This may be attributed to the progressive physicochemical deterioration of the coating during prolonged exposure to chloride ions, which can facilitate electrolyte penetration through the coating and promote corrosion reactions at the underlying metal surface. The Cu-sorbate alone system (CS-I with 20%) also shows more resistance up to the 15^th^ day, and then the resistance drops significantly. At the same time, increasing the CuO content (>10%) in the CS-III, CS-IV, and CS-V leads to a decrease in the resistance at a steady and constant rate, compared to CS-II. These results show that CuO plays a very crucial role in maintaining the stability of the coating in later immersion periods. From the electrochemical impedance studies, it was very clear that corrosion resistance was initially higher in the CS rich systems (CS-II and CS-I) up to 15 days, and decreased rapidly beyond the 15^th^ day. Besides, in the presence of more CuO with Cu-sorbate, resistance decreased very slowly with increasing immersion period.

#### 3.4.2 Potentiodynamic polarisation studies

The potentiodynamic polarisation studies were also carried out for all the coated specimens after 15 days of immersion time, and the data obtained from the Tafel analysis, such as current density (*I*_*corr*_), corrosion potential (*E*_*corr*_), anodic Tafel slopes (*β*_*a*_), and cathodic Tafel slopes (*β*_*c*_), were presented in Table 4 (Figure 9). Corrosion potential (*E*_*corr*_) was proportional to the corrosion reaction occurring in the systems. In general, a higher corrosion potential and a lower current density indicate higher corrosion resistance and barrier properties (Lu et al., 2017). According to the results of Table 4, the CS-II system has the highest *E*_*corr*_ and the lowest current density *I*_*corr*_ compared to other samples. An increase in the CuO content in the CS-III, CS-IV, and CS-V compositions led to a consistent decrease in their corrosion potential, as well as to an increase in the current density. After 30 days of immersion, a similar kind of trend was noticed in these systems: CS-rich paints (CS-II and CS-I) showed much less *I*_*corr*_ than the CuO-rich paint system. Further, these observations confirm that stable corrosion resistance was expressed in the copper sorbate-rich (CS-II and CS-I) coated surfaces, and resistance was decreased significantly in the coatings having less copper sorbate and higher CuO. The bare MS exhibited the lowest potential among all tested systems, which confirms its high susceptibility to the NaCl medium.

**TABLE 4:** Potentiodynamic polarisation studies of different anti-corrosive paints coated specimens immersed in 3.5% NaCl at different time intervals.

Paint name	Day	E_corr_ (mV)	I_corr_ (µA)	β_a_ (mV)	β_c_ (mV)
CS -I	15	−482.10	29.71	208.9	543.0
	30	−571.14	38.64	245.6	486.4
CS-II	15	−380.75	29.38	264.5	399.1
	30	−459.50	30.67	273.5	437.6
CS-III	15	−493.99	52.02	258.0	498.9
	30	−575.90	55.15	227.6	460.9
CS-IV	15	−614.33	59.01	211.9	548.6
	30	−613.18	78.55	149.5	325.8
CS- V	15	−611.48	121.05	211.7	649.6
	30	−631.55	144.01	208.5	531.7
Bare	15	−846.72	299.60	264.7	157.8
	30	−822.25	180.62	209.0	108.7

*E*_*corr*_ - corrosion potential, *I*_*corr*_ - current density, *β*_*a*_ - anodic Tafel slopes, and *β*_*c*_ - cathodic Tafel slopes.

**FIGURE 9:**
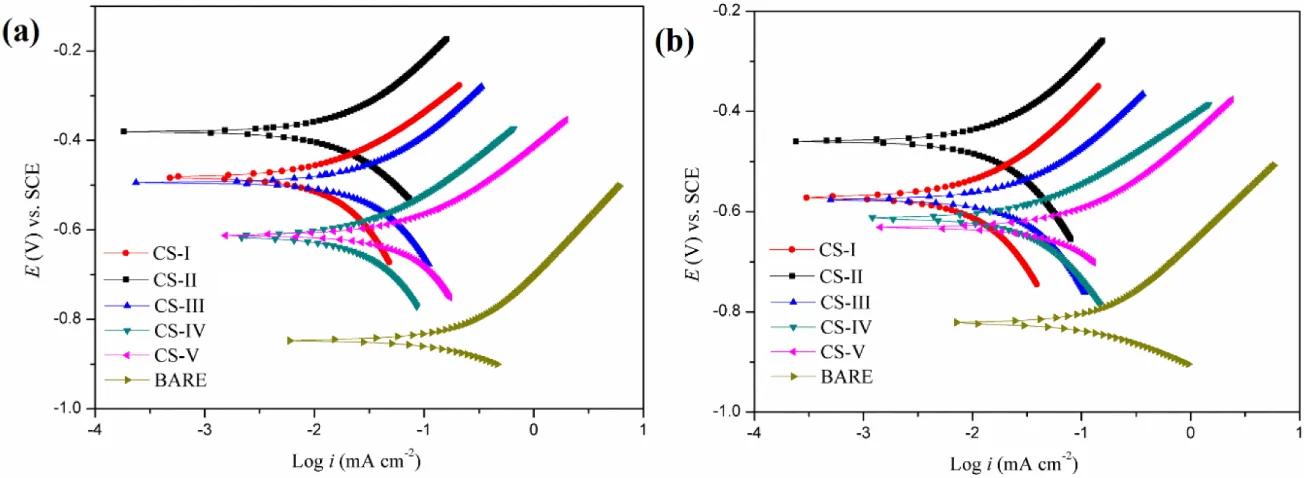
Potentiodynamic polarisation curves of different anti-corrosive paint-coated specimens immersed in 3.5% NaCl at various time intervals **(a)** 15th Day, and **(b)** 30^th^ Day.

### 3.5 Salt spray studies

The anticorrosion activity of the formulated paints was evaluated by simulating a field atmosphere using a salt spray chamber, and the digital observations were presented in Figure 10. A 3.5% NaCl solution was continuously sprayed up to 30 days using compressed air, and periodic changes that occurred on the surface of the coatings were visually observed. After starting to expose in a salt environment, rust formation was noticed in bare MS (uncoated) within 24 h; the remaining paints were unchanged for the initial days. After 5 days of exposure, slight changes were observed in CS-IV and CS-V. This may be because chloride ions start to react with CuO present in those paints. Similar observations were noticed in CS-III after the 15^th^ day of salt fog exposure. Very slight changes, such as the original colour being slightly faded and minimal blistering on the surface of CS-II, were observed. No major changes were observed in the CS-I system even at the end of the 30^th^ day.

**FIGURE 10:**
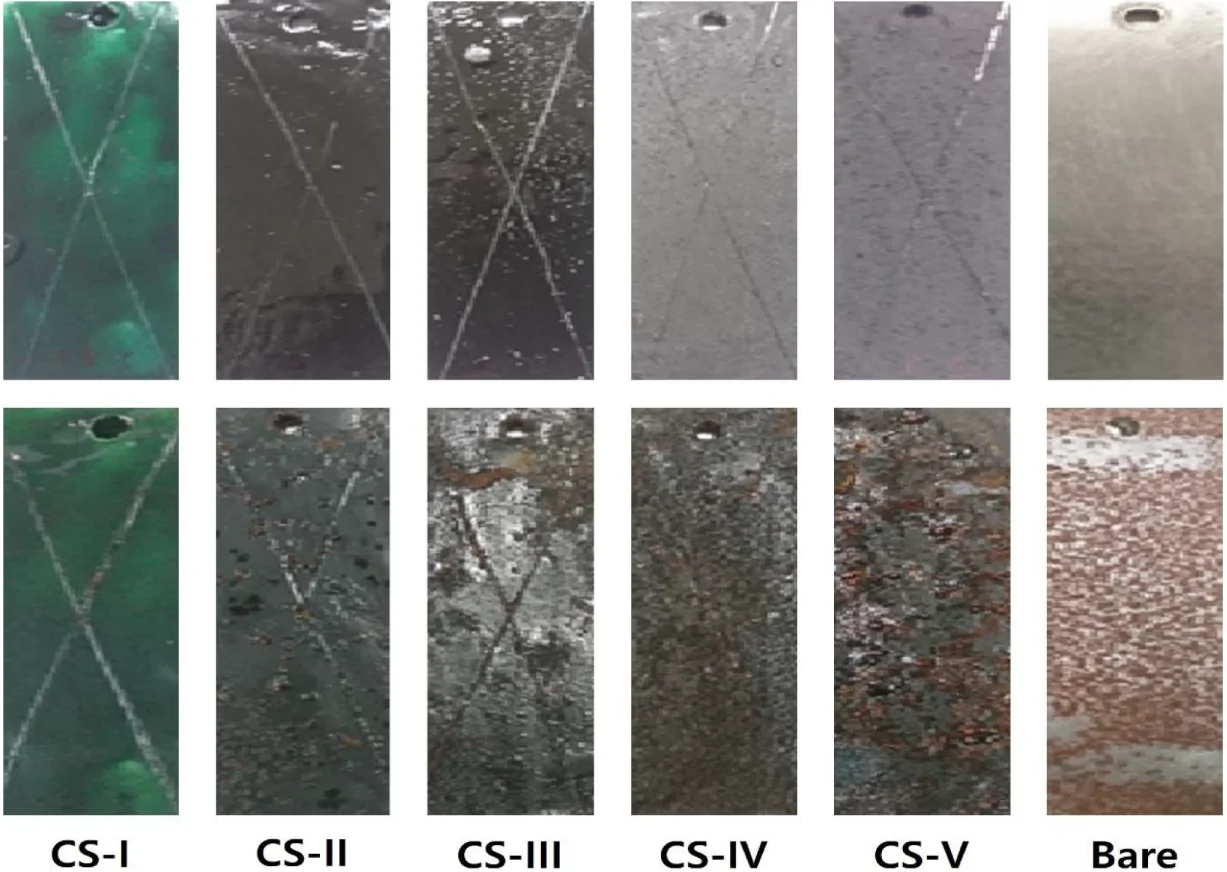
Salt spray (fog) exposure on different anti-corrosive paints coated specimens: top row before exposure; bottom row after the 30th day.

At the end of the exposure, it was clearly noticed that CS-V paint was highly damaged or changed from its original state; this may be due to the higher reactivity of CuO. Both the CS-I and CS-II systems show better corrosion resistance than other coated surfaces. This may be due to the lower reactivity of sorbate material with chloride ions. From the salt spray study, it was very clear that the anti-corrosion capability of the paints was increased with an increase in the concentration of CS in the following order: I > II > III > IV > V.

### 3.6 Anti-fouling studies

After completing the anti-corrosion study, marine antifouling studies were conducted for all the coated specimens by immersing them in estuary water, where vigorous fouling attack was a common phenomenon. During the immersion study, all the specimens were visually observed at regular intervals and digitally recorded and presented in Figure 11. After 30-day exposure, microorganisms started to settle over the bare MS steel and formed a very dense biofilm over the surface. This is probably due to the settlement of marine-biofilm forming bacterial species, since the colour of the biofilm was yellowish-orange. Visually, no slime was noticed in any of the paint-coated specimens; only some physiological changes were noticed. It might be due to the water current and dissolution of the seawater over the coatings, or it may be due to the leaching effects. Similarly, all the immersed specimens were observed on the 60^th^ day of the immersion period, and it was clear that the biofilm formed over the bare MS was much thicker and denser. Apart from that, very low slime content was observed over the surface of CS-I and CS-II, which indicates the beginning of the settlement of micro-fouling organisms over the coated specimen. For the CS-III specimen, much denser slime was noticed over the surface, which might be due to the settled microbial colonies producing extra-cellular polymeric substances (EPS). A notable rusted region was observed in the CS-V coating. This may be due to the pinhole formed during the coating. It was clear that the coating started to damage or fail in the field conditions.

**FIGURE 11:**
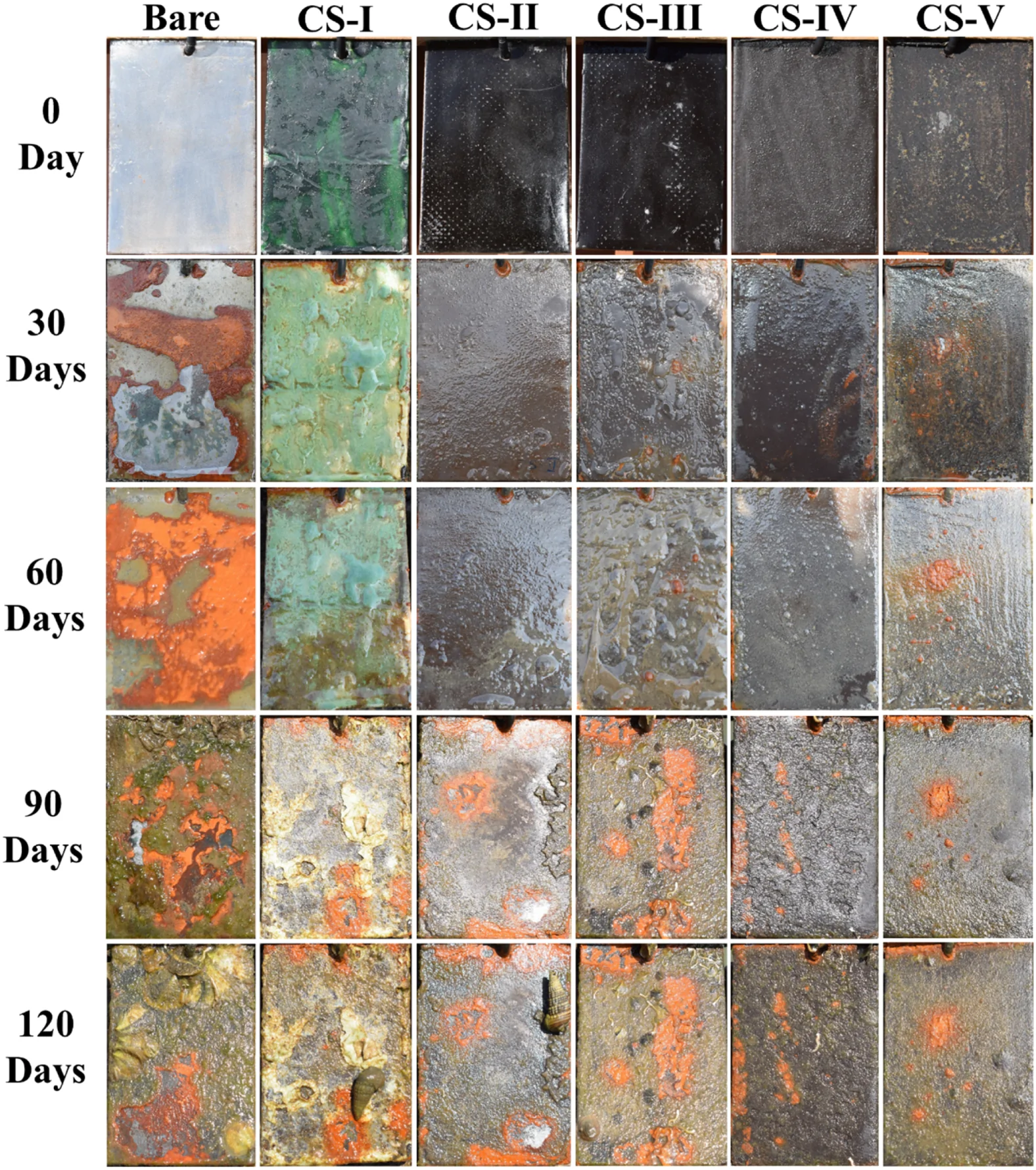
Digital photographs of the field immersion study of different coatings at different time intervals.

After the 90^th^ day of immersion, all the coated specimens were visualised, and found that bare MS had undergone a massive fouling attack with macro-fouler settlement throughout the surface. The Cu-sorbate pigment appears to have undergone moderate leaching from CS-I during prolonged seawater exposure. Still, the specimen exhibited limited corrosion and fouling attack, suggesting that a sufficient amount of the active pigment remained within the coating to contribute to its antifouling performance. Slight rust was observed on the edges of CS-II, and the orange-coloured rust appeared in the middle of CS-III; the remaining coupons, CS-IV and CS-V, were not fouled that much and were also not corroded except for a small pit in CS-V. It may be due to the toxic effects of CuO against a broad range of marine fouling organisms. After the 120^th^ day of immersion, all the specimens were observed for their antifouling susceptibility, and found that micro and macro-fouling organisms were fully occupied on bare MS. Also, some of the seashells started to attach to the painted surface of CS-I, CS-II, and CS-III. A white cylindrical tube-like structure was also formed over CS-III and CS-IV, along with green algae settlement. The major outbreaks in these antifouling paints were only a few types of macro-fouling organisms that started to appear after 120th days of immersion without the dominance of barnacles, oysters, mussels, hydroids, and bryozoans. This outcome was due to the foul release, which may harm and prevent their attachment to the painted coatings. Based on the fouling resistance and attachment of macro-fouling organisms, painted coatings were ordered as CS-V > CS-IV > CS-II > CS-I > CS-III.

### 3.7 SEM analysis of immersed specimens

During the 120 days of field immersion study, paint-coated MS coupons (1 cm^2^) placed for each respective coating were retrieved and subjected to SEM analysis, and their lower to higher magnifications of SEM images are shown in Figure 12. Very interesting changes were observed in every coating. Overall, microorganisms were observed in all the immersed coated and uncoated specimens. The only difference in the degree of attack and diversity of microorganisms was found in each system. In an uncoated substrate (Figure 12a), very vast and diverse microbial species were observed, which may be due to the settlement of free-floating microorganisms such as microalgae and bacterial colonies. It was clearly noticed that many of the phytoplankton, especially a diverse group of diatoms, had settled over the uncoated substrate (Sabbe et al., 2019). Paint CS-I was covered by one or two types of diatoms (Figure 12b). In the case of CS-II, the surface was changed roughly, and the smallest number of microbial cells was noticed (Figure 12c). This may be due to its anti-fouling capability over diverse organisms. In Figure 12d, very diverse colonies and species were observed in the case of CS-III paint, which was proven from visual observation as well. A uniform colonisation by a single species (belonging to phytoplankton) was observed over the surface of CS-IV paint, over the surface of CS-IV paint (Figure 12e), which illustrates that the paint was also prone to micro-fouling attacks. In Figure 12f, CuO paint was shown to visually show slime formation, which was proven from observation of some diverse cells, including a group of diatoms (Chen et al., 2019).

**FIGURE 12:**
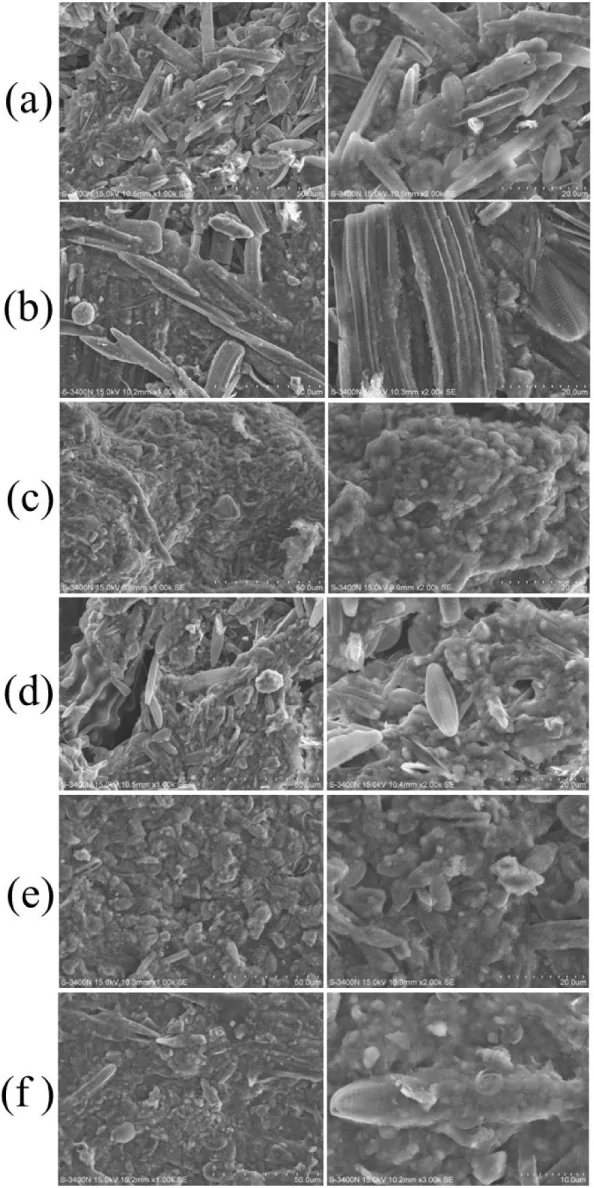
SEM analysis of coatings surfaces collected after 120 days of marine field immersion exposure: **(a)** Bare MS, **(b)** CS-I, **(c)** CS-II, **(d)** CS-III, **(e)** CS-IV, and **(f)** CS-V.

From this surface analysis, it was very clear that slime formation was not observed up to the 60^th^ day on immersed coatings. Afterwards, free-floating microorganisms were attached to the coating and started to develop and multiply, which was highly detrimental to coatings CS-I, CS-III, and CS-V. Further, these attachments may contribute to biofilm development on the coatings; however, only slow colonization was observed. This may be due to the leaching of the pigment from the coating, which suppresses many of the microorganisms. This observation strongly supports that copper sorbate-rich CuO paint (CS-II) suppresses the micro-foulers up to 120 days. This leached pigment well controlled the settlement of the majority of macro-fouling groups as well. The developed copper sorbate-based antifouling coating is primarily designed for submerged marine applications, including ship hulls, boat bottoms, offshore structures, aquaculture cages, seawater pipelines, and other immersed metallic infrastructures where biofouling and corrosion can adversely affect operational performance and service life.

### 3.8 Anti-fouling and anti-corrosion mechanism by copper sorbate and copper oxide paints

Copper sorbate was synthesized along with copper oxide as an antifouling pigment. Alkyd resin was used as a binder because of its film-forming, adhesion, elasticity, and compatibility. CS-II showed better corrosion resistance compared to other systems, which consist of 5% CuO with 15% copper sorbate. This anticorrosion activity was due to the synergistic action of the incorporated copper pigments. During the marine immersion, controlled leaching of copper species from both pigments (including CS-IV (15% CuO and 5% copper sorbate)) maintains continuous biocidal activity, which must decrease the initial attachment of microorganisms. At the same time, the alkyd binder acts as an effective barrier against corrosive species present in the seawater, minimizing corrosion reactions at the surface of mild steel. This can be confirmed from the higher impedance and lower corrosion current density of CS-II after 30 days. Visual observations from the salt spray study, marine immersion study, and immersed specimens’ SEM analysis strongly support that combined biocidal action and corrosion protection effectively minimize surface deterioration that otherwise promotes fouling, resulting in long-term protection under marine exposure.

## 4 Conclusion

In summary, copper sorbate pigment was successfully synthesized and utilized to develop alkyd-based anti-corrosive and antifouling coatings with varying concentrations of copper sorbate and CuO. The formulated paints and their coated substrates were evaluated through various physical characterization tests, including cross-hatch adhesion, bending, and fineness measurements. These tests confirmed that the coatings exhibit good adhesion and flexibility on the substrate. The SEM analysis revealed that the coatings were uniform and smooth. The electrochemical impedance spectroscopy confirms higher charge transfer resistance in the copper sorbate-rich CS-II system. However, these higher resistances gradually decreased with increasing immersion time. Bode plots, including impedance and phase angle analyses, further support the enhanced anti-corrosion behavior of the coatings. Potentiodynamic polarization studies demonstrated that the CS-II coating exhibits a higher corrosion potential and significantly lower corrosion current density compared to other formulations. These findings are consistent with the results obtained from salt spray testing, where the CuO-rich coating (CS-V) showed pronounced corrosion, evidenced by visible rust formation and surface degradation under salt fog exposure. Antifouling performance was evaluated by immersing the coated specimens in estuarine marine water. Rapid fouling was observed on bare mild steel (MS), whereas all coated samples exhibited improved resistance to both micro- and macro-fouling organisms. The immersion studies indicate that the presence of Cu-sorbate and CuO in the alkyd matrix contributed to the inhibition of fouling-organism settlement, with effective antifouling performance observed for up to 90 days. However, prolonged immersion for 120 days resulted in partial coating degradation and a decline in antifouling efficiency. The present study demonstrates the antifouling potential of copper sorbate-based coatings under long-term marine exposure conditions. However, detailed mechanistic studies involving biofilm characterization, microbial community analysis, copper-release kinetics, and ecotoxicological evaluation need to be thoroughly investigated to apply this antifouling coating at a further level.

## Data Availability

The raw data supporting the conclusions of this article will be made available by the authors, without undue reservation.
