# Supplementary material for: Development of alkyd-based copper sorbate coatings for anti-corrosion and anti-biofouling applications
Source: Front Chem. 2026 Sep 30;14:1838832. doi: 10.3389/fchem.2026.1838832 (PMC13661454; doi:10.3389/fchem.2026.1838832)
Supplement: Supplementary file 1 [file DataSheet1.docx]

**Supplementary Information**

**Development of alkyd-based copper sorbate coatings for anti-corrosion and anti-biofouling applications**

Manikandan Thiyagarajan^a^, Subramania Angaiah,^a,b^*, Chao Yan^a^*, Parthipan Punniyakotti^c^*

^a^School of Materials Science and Engineering, Jiangsu University of Science and Technology, Zhenjiang, 212003, P. R. China

^b^Electro-Materials Research Laboratory, Centre for Nanoscience and Technology, Pondicherry University, Puducherry-605014, India.

^c^Department of Biotechnology, Faculty of Science and Humanities, SRM Institute of Science and Technology, Kattankulathur, Chengalpattu, Tamil Nadu, 603203, India.

^*^**Corresponding authors:**

*Prof. Chao Yan (E-mail:* [*chaoyan@just.edu.cn*](mailto:chaoyan@just.edu.cn)*)*

*Prof. Subramania Angaiah (E-mail:* [*a.subramania@gmail.com*](mailto:a.subramania@gmail.com))

*Dr. Parthipan Punniyakotti (E-mail:* [*pparthibiotech@gmail.com*](mailto:pparthibiotech@gmail.com)*; parthipp@srmist.edu.in )*

**Fig. S1.** Fineness test of formulated paints.

**Fig. S2.** Cross hatch cut test of different coated specimens.

**Fig. S3.** Conical mandrel bend test of different coatings shows good flexibility with no cracks.
